# Mechanisms and therapeutic applications of electromagnetic therapy in Parkinson’s disease

**DOI:** 10.1186/s12993-015-0070-z

**Published:** 2015-09-07

**Authors:** Maria Vadalà, Annamaria Vallelunga, Lucia Palmieri, Beniamino Palmieri, Julio Cesar Morales-Medina, Tommaso Iannitti

**Affiliations:** Department of General Surgery and Surgical Specialties, University of Modena and Reggio Emilia Medical School, Surgical Clinic, Modena, Italy; Department of Medicine and Surgery, Centre for Neurodegenerative Diseases (CEMAND), University of Salerno, Salerno, Italy; Department of Nephrology, University of Modena and Reggio Emilia Medical School, Surgical Clinic, Modena, Italy; Centro de Investigación en Reproducción Animal, CINVESTAV-Universidad Autónoma de Tlaxcala, Tlaxcala, Mexico; Department of Neuroscience, Sheffield Institute for Translational Neuroscience (SITraN), University of Sheffield, Sheffield, UK

**Keywords:** Parkinson’s disease, Electromagnetic therapy, Transcranial magnetic stimulation, Repetitive transcranial magnetic stimulation, High-frequency transcranial magnetic stimulation, Pulsed electromagnetic field therapy

## Abstract

Electromagnetic therapy is a non-invasive and safe approach for the management of several pathological conditions including neurodegenerative diseases. Parkinson’s disease is a neurodegenerative pathology caused by abnormal degeneration of dopaminergic neurons in the ventral tegmental area and substantia nigra *pars compacta* in the midbrain resulting in damage to the basal ganglia. Electromagnetic therapy has been extensively used in the clinical setting in the form of transcranial magnetic stimulation, repetitive transcranial magnetic stimulation, high-frequency transcranial magnetic stimulation and pulsed electromagnetic field therapy which can also be used in the domestic setting. In this review, we discuss the mechanisms and therapeutic applications of electromagnetic therapy to alleviate motor and non-motor deficits that characterize Parkinson’s disease.

## Background

### Parkinson’s disease

Parkinson’s disease (PD) is one of the most common neurodegenerative diseases worldwide, second only to Alzheimer’s disease (AD) [1]. PD is accompanied by the impairment of the cortico-subcortical excitation and inhibition systems, hence belonging to the involuntary movement diseases [2]. PD is caused by progressive loss of structure and function of dopaminergic neurons in the ventral tegmental area and substantia nigra *pars compacta* in the midbrain with subsequent damage to the basal ganglia (BG) [3]. Cumulative evidence supports the hypothesis that PD is the result of complex interactions among genetic abnormalities, environmental toxins and mitochondrial dysfunction [4–6]. The mechanisms of neuronal degeneration characterizing PD have been studied extensively and include a complex interplay among multiple pathogenic processes including oxidative stress, protein aggregation, excitotoxicity and impaired axonal transport [7]. The increasing number of genes and proteins critical in PD is unraveling a complex network of molecular pathways involved in its etiology, suggesting that common mechanisms underlie familial and sporadic PD, the two forms of this pathology. While the sporadic form is the most common (90–95% of PD cases), only 5–10% of PD cases are familial [8, 9]. At least ten distinct loci are responsible for rare Mendelian forms of PD and mutations in five genes have been linked to familial PD [10]. PD is characterized by motor and non-motor symptoms. The main motor symptoms include bradykinesia, tremor at rest (tremor affecting the body part that is relaxed or supported against gravity and not involved in purposeful activities [11]), rigidity and postural instability [12–17]. However, motor symptoms are now considered as the “tip of the iceberg” of PD clinical manifestations. PD non-motor symptoms include cognitive decline, decrease in sleep efficiency, increased wake after sleep onset, sleep fragmentation, and vivid dreams as well as neuropsychiatric symptoms such as depression and psychosis, [18–23]. Pain syndrome and autonomic dysfunctions have also been observed in PD patients [24–26].

### Neuroimaging and genes: towards a personalized medicine for Parkinson’s disease

Several research groups have begun to perform genome-wide association studies (GWAS) on data or index measures derived from brain images, with the final goal of finding new genetic variants that might account for abnormal variations in brain structure and function that increase the risk of a given disease. Numerous genes have been identified using GWAS and have been associated with PD. They include alpha-synuclein, vacuolar protein sorting-associated protein 35, human leukocyte antigen family, leucine-rich repeat kinase 2 and acid β-glucosidase [27–29]. Neuroimaging associates individual differences in the human genome to structural and functional variations into the brain. Van der Vegt and colleagues reported structural and functional brain mapping studies that have been performed in individuals carrying a mutation in specific PD genes including PARK1, PARK2, PARK6, PARK7, PARK8, and discussed how this “neurogenetics-neuroimaging approach” provides unique means to study key PD pathophysiological aspects [30]. In addition, neuroimaging of presymptomatic (non-manifesting) mutation carriers has emerged as a valuable tool to identify mechanisms of adaptive motor reorganization at the preclinical stage that may prevent or delay PD clinical manifestation [30]. Neuroimaging may be useful to study the effectiveness of electromagnetic therapy in PD patients.

### Available therapies for Parkinson’s disease

PD treatment includes the use of pharmacological agents such as the dopaminergic agent l-3,4-dihy-droxy-phenylalanine (Levodopa or l-dopa) and stereotactic brain surgery which are associated with numerous side effects [31]. For example, the on-and-off phenomenon includes profound diurnal fluctuations in the psychomotor state of PD patients treated with l-dopa [32]. Furthermore, l-dopa loses effectiveness over time and can induce motor fluctuations such as the “wearing off” effect and dyskinesia [33]. While l-dopa metabolites are neurotoxic [33], the search for alternate, non-dopaminergic therapies to overcome the l-dopa-induced side effects has positioned adenosine A2A receptor (A2AR) antagonists as a promising therapeutic option for PD treatment [34]. Despite the favorable features of A2AR antagonists, their pharmacological properties, such as poor oral bioavailability and the lack of blood–brain barrier permeability, constitute a major problem to their clinical application [35]. Furthermore, regular physiotherapy and instrumental rehabilitation that have been employed to manage PD symptoms, such as tremor, slowness and difficulty in walking, are only moderately helpful [36]. Electromagnetic therapy has also been extensively used for PD treatment and may represent a promising therapeutic option for this condition since it promotes a lasting improvement in motor and non-motor symptoms [37–41].

### Electromagnetic therapy background

Electromagnetic therapy includes the use of six groups of electromagnetic fields as previously described [42, 43] and summarized below:

- *Static*/*permanent magnetic fields* can be created by various permanent magnets as well as by passing direct current through a coil.
- *Transcranial magnetic stimulation* (TMS) utilizes frequencies in the range 1–200 Hz.
- *Low*-*frequency electromagnetic fields* mostly utilize 60 Hz (in the US and Canada) and 50 Hz (in Europe and Asia) frequencies in distribution lines.
- *Pulsed radiofrequency fields* utilize frequencies in the range 12–42 MHz.
- *Millimeter waves* refer to very high-frequency in the range 30–100 GHz.
- *Pulsed electromagnetic fields* (*PEMFs*) utilize frequencies in the range 5–300 Hz with very specific shapes and amplitudes.

Electromagnetic therapy is defined as the use of time-varying electromagnetic fields of low-frequency values (3 Hz–3 kHz) that can induce a sufficiently strong current to stimulate living tissue [44]. Electromagnetic fields can penetrate all tissues including the epidermis, dermis, and subcutaneous tissue, as well as tendons, muscles and bones [45]. The amount of electromagnetic energy used and its effect on the target organ depends on the size, strength and duration of treatment [44]. Electromagnetic fields can be divided into two categories: static and time-varying. Electromagnetic therapy falls into two categories: (1) hospital use which includes TMS, repetitive transcranial magnetic stimulation (rTMS) and high-frequency TMS and (2) home use including PEMF therapy.

## Aim and searching criteria

We searched Pubmed/Medline using the keywords “Parkinson’s Disease” combined with “electromagnetic therapy”, “TMS”, “rTMS”, “high-frequency TMS” or “PEMF” and we included articles published between 1971 and 2015. This article aims to review the state of the art of electromagnetic therapy for treatment of PD.

## Transcranial magnetic stimulation

TMS is a safe and non-invasive method of electrical stimulation of neurons in the human cerebral cortex, modifying neuronal activity locally and at distant sites when delivered in series of pulses [46]. TMS is also a useful tool to investigate various aspects of human neurophysiology, particularly corticospinal function, in health and disease [47]. An electromagnetic field generator sends a current with a peak amplitude of about 8,000 A that lasts about 1 ms, through an induction coil placed on the scalp [48]. TMS is based on the principle of electromagnetic induction, as discovered by Faraday in 1838. The current flowing briefly in the iron coil placed over a patient’s head generates an electromagnetic field that penetrates the scalp and skull reaching the brain where it induces a secondary ionic current. The site of stimulation of the brain is the point along its length at which sufficient current passes through its membrane to cause depolarization [49]. TMS can be used to determine several parameters associated to different aspects of cortical excitability: (1) the resting motor threshold or active motor threshold which reflects membrane properties; (2) the silent period, which is a quiescent phase in the electromyogram (EMG), is partially of cortical origin and is related to the function of gamma-aminobutyric acid receptors; (3) the short intracortical inhibition and facilitation which occur when a subthreshold stimulus precedes a suprathreshold stimulus by less than 5 ms or 8–30 ms, respectively. The peak of electromagnetic field strength is related to the magnitude of the current and the number of turns of wire in the coil [50]. The electrical current is rapidly turned on and off in the coil through the discharge of electronic components called the capacitors.

### Transcranial magnetic stimulation in Parkinson’s disease

TMS clinical applications were first reported by Barker and colleagues who stimulated the brain, spinal cord and peripheral nerves using TMS with low or no pain [51]. Following this work, several TMS protocols that evidenced the correlation of TMS with peripheral EMG and monitored the modulation of TMS-induced motor evoked potentials (MEPs), were described [52–54]. For example, Cantello and coworkers studied the EMG potentials evoked in the bilateral first dorsal interosseus muscle by electromagnetic stimulation of the corticomotoneuronal descending system in 10 idiopathic PD patients without tremor but with rigidity with asymmetric body involvement and 10 healthy controls [55]. The threshold to cortical stimulation measured on the rigid side of PD patients was lower than on the contralateral side or than normal values. PD patients’ MEPs on the rigid side were larger compared to controls when the cortical stimulus was at rest or during slight tonic contraction of the target muscle [55]. Several clinical trials have pointed out the therapeutic efficacy of TMS in PD patients [3, 31, 56, 57]. For example, biomagnetic measurements performed using magnetoencephalography (MEG) in 30 patients affected by idiopathic PD exposed to TMS evidenced that 60% of patients did not exhibit tremor, muscular ache or dyskinesias for at least 1 year after TMS therapy [58]. The patients’ responses to TMS included a feeling of relaxation, partial or complete disappearance of muscular ache and l-dopa-induced dyskinesias as well as rapid reversal of visuospatial impairment [58]. Additional MEG measurements in PD patients also showed abnormal brain functions including slowing of background activity (increased theta and decreased beta waves) and increased alpha band connectivity [59]. These changes may reflect abnormalities in specific networks and neurotransmitter systems, and could be useful for differential diagnosis and treatment monitoring.

## Repetitive transcranial magnetic stimulation

rTMS is a non-invasive technique of brain stimulation based on electromagnetic induction [60]. rTMS has the potential to alter cortical excitability depending on the duration and mode of stimulation [61]. The electromagnetic pulse easily passes through the skull, and causes small electrical currents that stimulate nerve cells in the targeted brain region [62]. Since this type of pulse generally does not reach further than two inches into the brain, it is possible to selectively target specific brain areas [62]. Generally, the patient feels a slight knocking or tapping on the head as the pulses are administered. rTMS frequencies of around 1 Hz induce an inhibitory effect on cortical excitability [63] and stimulus rates of more than 5 Hz generate a short-term increase in cortical excitability [64]. rTMS induces a MEP of the muscles of the lower extremities by stimulating the motor and supplementary motor area (SMA) of the cerebral cortex [31].

### Repetitive transcranial magnetic stimulation in Parkinson’s disease

Several studies have reported the efficacy of rTMS on PD motor symptoms [65–69]. These effects are primarily directed at surface cortical regions, since the dopaminergic deficiency in PD is localized to the subcortical BG. The BG comprises a group of interconnected deep brain nuclei, i.e. the caudate and putamen, globus pallidus, *substantia nigra* and the subthalamic nucleus (STN) that, through their connections with the thalamus and the cortex, primarily influence the involuntary components of movement and muscle tone [70]. Several studies have documented the long-term effects of rTMS applied to PD patients for several days, rather than single sessions [71–73]. For instance, Shimamoto and coworkers applied rTMS on a broad area including the left and right motor, premotor and SMAs in nine PD patients for a period of 2 months, and observed improvements in the Unified Parkinson’s Disease Rating Scale (UPDRS), a rating scale used to follow PD progression [74]. A further trial in PD patients reported a shortened interruption of voluntary muscle contraction, defined cortical silent period, suggesting a disturbed inhibitory mechanism in the motor cortex [57]. PD patients show altered activation patterns in the SMA and overall less cortico-cortical excitability [75–81] that play a key role in motor selection in sequentially structured tasks, including handwriting. In a randomized controlled trial with a crossover design in PD patients, rTMS applied over the SMA influenced several key aspects of handwriting, e.g. vertical size and axial pressure, at least in the short term [82]. Ten PD patients treated with rTMS, evidenced short-term changes in functional fine motor task performance. rTMS over the SMA compensated for cortico-striatal imbalance and enhanced cortico-cortical connections. This treatment improved PD patients deficits such as reduction in speed during the writing task and decrease in letter size (micrographia).

Two mechanisms have been proposed to explain how cortically directed rTMS may improve PD symptoms: (1) rTMS induces brain network changes and positively affects the BG function; (2) rTMS directed to cortical sites compensates for PD-associated abnormal changes in cortical function [60]. Indeed, in support of the former mechanism, rTMS might modulate cortical areas, such as the prefrontal cortex and primary motor cortex, which are substantially connected to both the striatum and STN via glutamatergic projection, and thus indirectly modulate the release of dopamine in the BG [83]. Several TMS/functional imaging studies have demonstrated the effects of rTMS on BG and an increase in dopamine in the BG after rTMS applied to the frontal lobe [84].

rTMS can also transiently disrupt the function of a cortical target creating a temporary “virtual brain lesion” [85–87]. Mottaghy and coworkers have studied the ability of rTMS to produce temporary functional lesions in the BG, an area involved in working memory, and correlated these behavioral effects with changes in regional cerebral blood flow in the involved neuronal network [88]. Functional imaging and TMS studies in PD subjects have shown altered cortical physiology in areas associated to the BG such as the SMA, dorsolateral prefrontal cortex and primary motor cortex [57, 89], characterized by excessive corticospinal output at rest, concomitant to, or resulting from a reduced intracortical inhibition [60]. These altered changes in cortical function in PD patients might avoid the suppression of competing motor areas and therefore decrease the motor system performance, resulting in symptoms such as tonic contractions and rigidity [89].

rTMS has not only been applied to a motor area of the brain but has also been used to target PD non-motor deficits. For example, in a study involving six PD patients with mild cognitive impairment, a cognitive dysfunction defined by deficits in memory, rTMS was delivered over the frontal region at 1.2 times the motor threshold (minimum stimulation intensity) of the right abductor pollicis brevis muscle [3]. Over a period of 3 months, rTMS was performed for a total of 1200 stimulations. Improvement in neuropsychological tests (the trail-making test part B and the Wisconsin card-sorting test) was observed in all patients. In addition, an improvement in subjective symptoms and objective findings were also observed by the subjects, their families, and the therapists. The changes observed in PD subjects included “faster reactions”, “better body movement and smoother standing-up and movement”, “more active”, “more cheerful”, and “more expressive”. An increase in the amount of conversation, an increase in the neural mechanisms of mutual understanding within daily living and an improvement in responses to visitors were also noted, if compared to baseline. Additionally, changes such as better hand usage while eating and better sleep were also observed.

Cognitive dysfunction is often seen in PD patients with major depression and its neural basis could be the functional failure of the frontostriatal circuit [3, 90]. Ten days of rTMS in the frontal cortex can effectively alleviate PD-associated depression as shown by an open trial reporting a significant decrease in the Hamilton Depression Rating Scale (HDRS) scores [91]. A further double blind, sham stimulation-controlled, randomized study, involving 42 idiopathic PD patients affected by major or minor depression undergoing rTMS for 10 days, evidenced a mean decrease in HDRS and Beck depression inventory after therapy [92].

In opposition to the above mentioned positive reports concerning the efficacy of rTMS in PD patients, a lack of effectiveness of rTMS on objective or subjective symptoms has also been described. For example, in a study involving 85 idiopathic PD patients, no significant differences in clinical features were observed between patients receiving rTMS and sham stimulation [65]. Moreover, total and motor score of UPDRS were improved by rTMS and sham stimulation in the same manner. Despite this improvement, PD patients treated with rTMS revealed signs of depression, reporting no subjective benefits. In another randomized crossover study, 10 patients affected by idiopathic PD received rTMS to the SMA which resulted in subclinical worsening of complex and preparatory movement [93]. The rTMS protocol was not tolerated by 2 out of 10 patients. Furthermore, this study showed that, following rTMS, subtle regional disruption can persist for over 30 min, raising safety concerns. A further randomized crossover study involving 11 patients with idiopathic PD, treated with rTMS over the motor cortex, did not show any therapeutic effect on concurrent fine movement in PD [94].

In summary, conflicting findings regarding the efficacy of rTMS in PD have been reported and they can be explained by differences in stimulation parameters, including intensity, frequency, total number of pulses, stimulation site and total number of sessions. Therefore, further studies comparing different parameters are required.

## High-frequency transcranial magnetic stimulation

High-frequency TMS consists of continuous high-frequency stimulation of specific brain regions, including the motor cortex, cerebellum and BG, through implanted large four-contact electrodes connected to a pulse generator and positioned into the center of the target region [70]. Such stimulation induces an electrical field that spreads and depolarizes neighboring membranes of cell bodies, afferent and efferent axons, depending on neuronal element orientation and position in the field and on stimulation parameters [95]. Optimal clinical results are obtained by using pulses of 60–200 ms duration and 1–5 V amplitude, delivered in the STN at 120–180 Hz [96]. For example, high-frequency TMS produces a transient blockade of spontaneous STN activity, defined HFS-induced silence. During HFS-induced silence, the persistent Na^+^ current is totally blocked and the Ca^2+^-mediated responses are strongly reduced, suggesting that T- and L-type Ca^2+^ currents are transiently depressed by high-frequency TMS [97].

Indeed, recent evidence suggests that the stimulation of the motor cortex, the cerebellum and the BG not only produces inhibitory and excitatory effects on local neurons, but also influences afferent and efferent pathways. Therefore, the mechanism of action of high-frequency TMS depends on changes in neural activity generated in the stimulated, afferent and efferent nuclei of the BG and motor cortex [98].

### High-frequency transcranial magnetic stimulation in Parkinson’s disease

In the first PD patients treated with high-frequency TMS in 1993, motor symptoms, tremor, rigidity and akinesia improved significantly allowing to decrease the administration of l-dopa by a mean of 55% [99]. Since then, several thousands of patients worldwide have been fitted with high-frequency TMS implants achieving marked improvements in their symptoms, making this method the reference procedure for advanced PD [100]. The time course of improvement following high-frequency TMS treatment differs for different cardinal symptoms of PD [101]. For instance, rigidity and resting tremor decrease immediately, within a few seconds after high-frequency TMS [102]. Different clinical effects are observed in PD patients depending on the site of stimulation [103]. For example, stimulation of the ventral intermediate nucleus of the thalamus can dramatically relieve PD-associated tremor [104]. Similarly, stimulation of the STN or globus pallidus interna (GPi) can substantially reduce rigidity, tremor, and gait difficulties in patients affected by idiopathic PD [105]. Stimulation of the GPi also reduces all of the major PD motor manifestations, including the reduction of l-dopa-induced dyskinesias and involuntary movements produced by individual doses of dopaminergic medications that can limit treatment efficacy [106]. Thalamic stimulation in the region of the ventral intermediate nucleus reduces limb tremor but it has little effect on other manifestations of the disease [107]. In order to explain the beneficial effects of high-frequency TMS, two fundamental mechanisms have been proposed by Garcia and coworkers: silencing and excitation of STN neurons [95]. They reported that high-frequency TMS using stimulus parameters that yield therapeutic effects has a dual effect, i.e. it suppresses spontaneous activity and drives STN neuronal activity. High-frequency TMS switches off a pathological disrupted activity in the STN (i.e. silencing of STN neurons mechanism) and imposes a new type of discharge in the upper gamma-band frequency (60–80 Hz range) that is endowed with beneficial effects (i.e. excitation of STN neurons mechanism) [95]. This improvement generated by high-frequency TMS is due to parallel non-exclusive actions, i.e. silencing of ongoing activity and generation of an activity pattern in the gamma range [108]. There is an important advantage in silencing spontaneous activity and generating a pattern: the signal to noise ratio and the functional significance of the new signal are enhanced [109].

Techniques and preparations employed to study the mechanisms of high-frequency TMS include electrophysiological techniques, measurement of neurotransmitter release in vivo, post-mortem immunohistochemistry of a metabolic marker such as cytochrome oxidase and imaging studies in vivo [95]. Such results consistently show a post-stimulus period of reduced neuronal firing followed by the slow recovery of spontaneous activity. High-frequency TMS, at frequencies >50 Hz, applied to the STN of PD patients undergoing functional stereotactic procedures [110–112], to the STN of rats in vivo [113, 114] and rat STN slices in vitro [97, 115, 116], produces a period of neuronal silence of hundreds of milliseconds to tens of seconds. During brief high-frequency TMS in PD patients off medication and in the murine model of parkinsonism obtained by acute injections of neurotoxin 1-methyl-4-phenyl-1,2,3,6-tetrahydropyridine for 5 consecutive days, a reduced STN activity, as response to stimulation, is observed at 5–14 Hz and this response is frequency-dependent [114]. High-frequency TMS has two main advantages: (a) it reduces the time a patient spends in the “off” state because the individual dose of these profound diurnal fluctuations leaves a person slow, shaky, stiff, and unable to rise from a chair; (b) it allows the reduction of medications and their consequent side effects [117].

## Pulsed electromagnetic field therapy

PEMF therapy is a non-static energy delivery system, characterized by electromagnetic fields inducing microcurrents in the target body tissues [118]. These microcurrents elicit specific biological responses depending on field parameters such as intensity, frequency and waveform [119]. The benefits of PEMF therapy have been observed in several clinical studies for treatment of several medical conditions including knee osteoarthritis [120], shoulder impingement syndrome [121], lower back pain [122, 123], multiple sclerosis [124, 125], cancer [121, 123, 125, 126], PD [127], AD [128] and reflex sympathetic dystrophy syndrome [129]. A large number of PEMF therapy devices contains user-friendly software packages with pre-recorded programs with the ability to modify programs depending on the patient’s needs [43, 130–132]. Examples of PEMF devices are the Curatron^®^ (Amjo Corp, West Chester, PA, USA), Seqex^®^ system (S.I.S.T.E.M.I. Srl, Trento, Italy), MRS 2000^®^, iMRS^®^, QRS^®^ (all produced by Swiss Bionic Solutions Schweiz GmbH, Dulliken, Switzerland) and TESLA Stym (Iskra Medical, Ljubljana, Slovenia).

### Pulsed electromagnetic field therapy in Parkinson’s disease

In October 2008 the Food and Drug Administration approved the use of PEMF therapy for treatment of major depressive disorder in PD patients who failed to achieve satisfactory improvement from very high dosages of antidepressant medications [133, 134]. Several studies reported PEMF therapy improved cognitive functions and motor symptoms. For example, an investigation involving three elderly PD patients with cognitive impairment assessed the effect of PEMF therapy on macrosomatognosia, a disorder of the body image in which the patient perceives a part or parts of his body as disproportionately large [135]. After receiving PEMF therapy, PD patients’ drawings showed reversal of macrosomatognosia (assessed by Draw-a-Person test) with reduction of the right parietal lobe dysfunction. Furthermore, PEMF therapy applied to a 49-year-old male PD patient with stage 3 disease, as assessed by Hoehn and Yahr scale, resulted in a marked improvement in motor and non-motor symptoms such as mood swings, sleeplessness, pain and sexual and cognitive dysfunctions, suggesting that PEMF therapy should be tested in large cohorts of PD patients as monotherapy and should also be considered as a treatment modality for de novo diagnosed PD patients [136]. PEMF therapy was also effective in improving visuospatial deficits in four PD patients, as assessed by the clock-drawing test [137]. Moreover, PEMF therapy improved PD-associated freezing (a symptom manifesting as a sudden attack of immobility usually experienced during walking) in 3 PD patients through the facilitation of serotonin neurotransmission at both junctional and non-junctional neuronal target sites [127].

## Discussion

Although many studies on electromagnetic therapy included only a small number of participants, several investigations suggest that this therapy is effective in treating PD patients’ motor and non-motor symptoms. In the development of electromagnetic therapies, it is important to clarify the pathophysiological mechanisms underlying the symptoms to treat in order to determine the appropriate brain region to target. Thus, in the future, electromagnetic therapy must tend towards a more personalized approach, tailored to the specific PD patient’s symptoms. All the types of electromagnetic therapy described in this review can be used in combination with pharmacological and non-pharmacological therapies but this approach is understudied in PD patients. Therefore, specific protocols should be designed and tested in combination with other therapies in future controlled trials in patients affected by PD.

### Transcranial magnetic stimulation

TMS increases the release of dopamine in the striatum and frontal cortex, which in turn improves PD symptoms including motor performance [138]. Furthermore, TMS applied in the prefrontal cortex induces the release of endogenous dopamine in the ipsilateral caudate nucleus as observed by positron emission tomography in healthy human subjects [89]. TMS application results in partial or complete disappearance of muscular pain and l-dopa-induced dyskinesia as well as regression of visuospatial impairment. This clinical improvement is followed by MEG improvement and normalization recorded after TMS, suggesting that TMS has an immediate and beneficial effect on corticostriatal interactions that play an important role in the pathophysiology of PD [58]. Cerasa and coworkers observed that repetitive TMS applied over the inferior frontal cortex reduced the amount of dyskinesia induced by a supramaximal single dose of levodopa in PD patients, suggesting that this area may play a key role in controlling the development of dyskinesia [139]. The mechanism underlying TMS effectiveness in PD remains an unanswered question due to the complexity of behavioral and neuroendocrine effects exerted by the TMS when applied to biological systems and their potential impact on neurotransmitter functions [140]. The effect of TMS differs depending on the stage of the disease, the age of disease onset, the amount of cerebral atrophy and genetic factors [37]. TMS has a low cost and is simple to operate and portable, opening the possibility for patients to perform at home stimulation which could be of high relevance in the elderly and in patients who are severely disabled. As far as side effects are concerned, the muscles of the scalp, jaw or face may contract or tingle during the procedure and mild headache or brief lightheadedness may occur [141, 142]. A recent large-scale study on the safety of TMS found that most side effects, such as headaches or scalp discomfort, were mild or moderate, and no seizures occurred [143]. Although evidence shows that TMS exerts complex cellular, systemic and neuroendocrine effects on biological systems impacting neurotransmitter functions [58], future controlled studies in larger cohorts of patients and with a long term follow-up are needed to further clarify the mechanisms underlying TMS efficacy in PD patients.

### Repetitive transcranial magnetic stimulation

rTMS can be defined as a safe and non-invasive technique of brain stimulation which allows to specifically treat PD with low-frequency electromagnetic pulses [60]. As opposed to high-frequency TMS, which can induce convulsions in healthy subjects, rTMS does not affect the electroencephalogram pattern [71, 144]. Slow waves have been induced by rTMS over the right prefrontal area, a brain area involved in executive dysfunction that is observed in early stages of PD and is characterized by deficits in internal control of attention, set shifting, planning, inhibitory control, dual task performance, decision-making and social cognition tasks [3, 145]. rTMS applied to PD patients, enhances not only executive function, but also motor function, subjective symptoms and objective findings [3]. rTMS also increases cognitive function and other symptoms associated to the prefrontal area in PD patients [146]. In PD patients, therapeutic efficacy and long-term benefits of rTMS are obtained following multiple regular sessions rather than single sessions, but side effects associated to this therapy still warrant investigation in large controlled trials.

### High-frequency magnetic stimulation

The observations that STN activity is disorganized in PD patients and that a lesion or chemical inactivation of STN neurons ameliorate motor symptoms led to the hypothesis that high-frequency TMS silences STN neurons and, by eliminating a pathological pattern, alleviates PD symptoms [147–151]. Garcia and colleagues proposed another hypothesis suggesting that high-frequency TMS suppresses not only the pathological STN activity but also imposes a new activity on STN neurons [95]. They proposed that high-frequency TMS excites the stimulated structure and evokes a regular pattern time-locked to the stimulation, overriding the pathological STN activity. As a consequence, high-frequency TMS removes the STN spontaneous activity and introduces a new and regular pattern that improves the dopamine-deficient network [95]. Elahi and coworkers found that high-frequency TMS modulates the excitability of the targeted brain regions and produces clinically significant motor improvement in PD patients [66]. This improvement is due to parallel non-exclusive actions, i.e. silencing of ongoing activity and generation of an activity pattern in the high gamma range [152]. Several clinical studies reported positive clinical results following high-frequency TMS in l-dopa-responsive forms of PD, including patients with selective brain dopaminergic lesions [153]. It remains unclear whether the mechanisms of action of high-frequency TMS and l-dopa are similar or they could be even synergic. However, high-frequency TMS improves the l-dopa-sensitive cardinal motor symptoms of PD patients with benefits similar to those given by l-dopa, though with reduced motor complications [154, 155]. The interactions with the dopaminergic system seem to be a key factor explaining the efficacy of both treatments [156]. High-frequency TMS changes dopamine lesion-induced functional alterations in the BG of PD animal models and gives an insight into the mechanisms underlying its antiparkinsonian effects [114, 157, 158]. The intrinsic capacity of the BG to generate oscillations and change rapidly from a physiological to a pathogenic pattern is crucial; the next step will be to identify how high-frequency TMS is propagated inside the BG. Disadvantages of this therapy are the high cost and limited availability of the devices to specialized medical centers, limited knowledge of potential long-term side effects and the necessity to employ highly trained personnel.

### Pulsed electromagnetic fields

PEMF therapy improves PD symptoms including tremor, slowness of movement and difficulty in walking [159]. It is non-invasive, safe and improves PD patients’ quality of life [124, 160]. PEMF therapy, employed for PD treatment, supports the body’s own healing process for 4–6 h after therapy session [161–163]. It can be used at home and applied to the entire body or locally to target a specific body area and, if compared with dopaminergic systemic therapy, e.g. l-dopa, it can offer an alternative treatment avoiding systemic side effects such as hepatotoxicity and nephrotoxicity.

## Conclusions

Electromagnetic therapy opens a new avenue for PD treatment. Each electromagnetic therapy technique described in this review can be applied according to a single protocol or as a combination of different protocols specifically tailored to the PD patient’s needs. Beyond the necessity to choose coil or electrode size and placement, there is a variety of parameters that have to be taken into account when designing electromagnetic therapy approaches and they include stimulation intensity, duration, frequency, pattern, electrode polarity and size. Furthermore, electromagnetic therapy can also be combined with pharmacological or non-pharmacological treatments, e.g. physical therapy and cognitive tasks, to produce additive or potentiated clinical effects. In conclusion, electromagnetic therapy represents a non-invasive, safe and promising approach that can be used alone or combined with conventional therapies for the challenging treatment of PD motor and non-motor symptoms.
